# Comparative Analysis of the Mitochondrial Genomes of Five Species of *Anabropsis* (Orthoptera: Anostostomatidae) and the Phylogenetic Implications of Anostostomatidae

**DOI:** 10.3390/biology14070772

**Published:** 2025-06-26

**Authors:** Tingting Yu, Siyu Pang, Wenjing Wang, Ting Luo, Yanting Qin, Xun Bian, Bin Zhang

**Affiliations:** 1 College of Life Sciences & Technology, Inner Mongolia Normal University, Hohhot 010022, China; yutt1284578337@163.com (T.Y.); 20211102787@mails.imnu.edu.cn (W.W.); 2 Key Laboratory of Ecology of Rare and Endangered Species and Environmental Protection (Guangxi Normal University), Ministry of Education, Guilin 541006, China; pangsiyu0820@outlook.com (S.P.); luot2024@163.com (T.L.); qinyanting2019@163.com (Y.Q.)

**Keywords:** Anostostomatidae, phylogenetic analysis, mitogenomics, Anabropsini

## Abstract

In China, Anostostomatidae is represented by a single tribe, Anabropsini; two genera; and 33 species. Although extensive research has been conducted on Anabropsini, the monophyly of this tribe within Anostostomatidae remains unverified. Furthermore, the phylogenetic relationships within *Anabropsis* remain under debate. To address these gaps, we sequenced and annotated the mitochondrial genomes of five Anabropsini species to investigate their mitochondrial characteristics and phylogenetic positions and clarify the relationships among *Anabropsis* subgenera. The total mitochondrial length of the five species ranged from 15,985 bp to 16,423 bp and contained 13 protein-coding genes, 22 tRNAs, 2 rRNAs, and 1 control region. A grouped analysis of selection pressure on *Anabropsis* revealed that the Ka/Ks values for alate and apterous forms are not significantly different, suggesting that using wing length alone as the basis for dividing subgenera within *Anabropsis* may be unreliable. Tertiary structure modeling of proteins showed that the variable sites were concentrated in α-helix regions. Phylogenetic trees were reconstructed using the Bayesian inference and maximum likelihood methods and were based on two better datasets, namely, PCG123 (all codon positions of the PCGs) and PCG123 + 2R (all codon positions of PCGs, 12SrRNA, and 16SrRNA). The results indicate that the Chinese Anabropsini is paraphyletic, whereas *Anabropsis* is monophyletic, with a stable subgeneric topology.

## Simple Summary

In China, the Anostostomatidae family is represented by a single tribe, Anabropsini, and two genera (*Anabropsis* and *Melanabropsis*). However, molecular data for Anabropsini remain limited, and its monophyly remains unverified. The phylogenetic relationships among the *Anabropsis* subgenera are also debated. To address these gaps, we sequenced and analyzed the mitochondrial genomes of five *Anabropsis* species. Additionally, we reconstructed the phylogeny of Anostostomatidae using maximum likelihood and Bayesian inference methods. The results indicated that Anabropsini is not monophyletic, and the topological structure between subgenera within the *Anabropsis* genus is stable.

## 1. Introduction

The subfamily Anostostomatidae Saussure, 1859 comprises 9 tribes and 33 genera. Among them, 1 tribe, 2 genera, and 33 species are found in China [1]. Most species are flightless nocturnal predators or omnivores, burrowing in the soil during the day to hide and emerging at night to feed or mate. Their diet includes other invertebrates, such as moths and grasshoppers, and fruits. Some species exhibit cannibalistic behavior in the wild. When threatened, some species release foul odors or, in some cases, enter water as a defensive strategy. While the loss of wings is a likely evolutionary scenario, they can also dig caves. In recent years, many new species have been discovered in China; however, most identifications have been based on morphology alone [2,3,4,5,6,7,8,9].

Rentz and Weissman established the Anabropsini Rentz & Weissman, 1973 tribe, with *Anabropsis* Rehn, 1901 designated as the type genus, based on species distributed in the Palearctic and Nearctic regions [10]. Later, Gorochov elevated Anabropsini to the subfamily status of Anabropsinae Rentz & Weissman, 1973 placing it within the Mimnermidae family [11]. He further established the genera *Pteranabropsis* Gorochov, 1988 and *Apteranabropsis* Gorochov, 1988 based on wing length. Gorochov observed greater morphological similarity among American species than between American and Asian taxa, and accordingly transferred Asian species to the *Pteranabresis* and *Apteranabropsis* genera while retaining American species under *Anabropsis* [11]. Gorochov hypothesized that the American Anabropsini tribe diverged later and may have originated in Asia [11,12].

Griffini distinguished between *Paterdecolyus* Griffini, 1913 and other Anabropsinae genera based on the absence of wings and the presence of fore tibiae with internal tympana [13]. Johns later revised the classification system of Anostostomatidae Saussure, 1859, after evaluating numerous specimens. He considered Mimnermidae Brunner von Wattenwyl, 1888 a synonym of Anostostomatidae and placed Anabropsini under Anostostomatidae [14]. He also considered *Pteranabropsis* and *Apteranabropsis* synonyms of *Paterdecolyus*, although without providing detailed justification [14]. In contrast, Gorochov argued that *Pteranabropsis* and *Apteranabropsis* may be synonyms of *Anabropsis*, and favored a classification system that separated species based on geographic distribution, assigning American, Asian, and other regional taxa into distinct genera [15].

Shi and Bian established the *Brevipenna* Shi & Bian, 2016 genus, which resembles *Pteranabropsis* as the wings only reach the middle of the abdomen [16]. *Pteranabropsis*, *Apteranabropsis*, and *Brevipenna* share similar features, except for obvious differences in wing length. Ingrisch argued that while the body size, wing length, wing surface, and length-to-width ratio vary among species, the tegminal venation and relation of the length of the anterior to that of the posterior area in the female subgenital plate can serve as more reliable diagnostic characters [17].

Gorochov restudied *Anabropsis* and proposed *Brevipenna* as a synonym of *Pteranabropsis* while reducing *Paterdecolyus*, *Apteranabropsis*, and *Pteranabropsis* to subgenera within *Anabropsis.* As a result, eight species from *Pteranabropsis* were transferred to *Anabropsis* (subgenus *Carnabropsis*) Gorochov, 2021 [18]. Pang et al. subsequently proposed two subgenera of *Apteranabropsis*, namely, *Spinanabropsis* Pang, Lu, & Bian, 2023 and *Pseudapteranabropsis* Pang, Lu, & Bian, 2023 [6]. Xu and Shi then reassigned *Anabropsis* (*Pseudapteranabropsis*) *nigrimacula* to *Anabropsis* (*Apteranabropsis*), based on the characteristics of male specimens [8]. However, as the number of Asian species increased, distinctions among the subgenera within the *Anabropsis* genus became increasingly ambiguous. Up to now, a total of 69 species of the genus *Anabropsis* have been discovered worldwide [1]. The extensive morphological diversity observed within the genus presents challenges in establishing stable monophyletic groups. Furthermore, hypotheses regarding the origin and evolution of these species, particularly those based on geographic distribution and wing length, remain speculative in the absence of molecular data [18,19].

The mitochondrial genome (mitogenome) is an effective marker for elucidating phylogenetic relationships and the evolutionary history of insect groups. The mitogenome is characterized by unique features, including low sequence recombination, maternal inheritance, and a fast-evolutionary rate [20,21]. In animals, the mitogenome represents a small, extrachromosomal, nearly circular molecule [22]. Animal mitochondrial genomes typically range from 15 to 20 kb in size and contain 37 genes, including 13 protein-encoding genes (PCGs), 2 mitochondrial ribosomal RNAs (rRNAs), 22 tRNAs essential for mitochondrial protein translation, and a control region (D-loop) [23]. Analyses of mitochondrial genome features, such as size, genome organization, and gene content arrangement, have proven to be powerful tools for inferring phylogenetic relationships among metazoans across various taxonomic levels [24]. Mitochondrial genome data have been used in population-level studies to phylum-level analyses [25,26].

Although mitochondrial genome data for Anostostomatidae were recently published, most studies have focused on mitochondrial genome structures, while relationships between subfamilies, genera, or intra-genera within Anostostomatidae remain unclear. Song et al. confirmed the monophyly of Anostostomatidae, based on whole mitochondrial and nuclear gene datasets [27]. Trewick et al. used 13 mitochondrial protein-coding genes and 4 nuclear protein-coding genes to verify that the New Zealand *Hemiandrus* Ander, 1938 does not form a monophyletic group and that the New Zealand and Australian *Hemiandrus* species are not sister taxa [28]. Lu published the complete mitochondrial genomes of five *Anabropsis* species, analyzed their mitochondrial genome characteristics, and validated the distinctiveness of certain species within the genus [2,3,4]. However, the monophyly of *Anabropsis* in China, according to this new genus division, has not been verified. Thus, the classification and subgeneric division within Anabropsis from China remains controversial.

In this study, we sequenced and annotated the five species (*A. (Pseudapteranabropsis) nigrimaculatis* Pang, Lu, & Bian, 2023; *A. (Spinanabropsis) erythronota* Pang, Lu, & Bian, 2023; *A. (Pseudapteranabropsis) flavimaculata* Pang, Lu & Bian, 2023; *A. (Apteranabropsis) daweishanensis* Pang, Lu & Bian, 2023 and *A. (Spinanabropsis) pengi* Pang, Lu & Bian, 2023) of the Chinese Anabropsini to clarify their evolutionary relationships. A phylogenetic tree was constructed based on the published mitochondrial genomes of Anostostomatidae to determine the phylogenetic position of Chinese Anabropsini within the family and to assess the monophyly of the group. This analysis aimed to elucidate the relationships among Anabropsini subgenera and evaluate the validity of wing length as a criterion for subgeneric classification.

## 2. Materials and Methods

### 2.1. Specimen Extraction and Sequencing

The specimens were collected in China (Table 1). They were immersed in 95% ethanol during collection and stored at −20 °C for long-term preservation in the Guangxi Normal University (Guilin, Guangxi, China). The total genomic DNA of five species was extracted from the muscle tissues of the hind leg, using TIANamp Genomic DNA kits (TIANamp, Beijing, China). DNA samples were sent to Berry Genomics (Beijing, China) for the Illumina HiSeq 2500 platform with paired reads of 2 × 150 bp to obtain the original data after quality control.

### 2.2. Mitochondrial Genome Sequence Assembly and Analysis

Raw paired-end reads were processed using CLC Genomics Workbench 12 to obtain high-quality clean reads. These reads were compared with the whole mitochondrial genome sequences of Anabropsinae species available in the NCBI database to determine the most homologous sequences, which would act as the reference genome (Table S1) [29]. Mitochondrial assembly was performed using NOVOPlasty 4.1.2. The MITOS2 (Galaxy) tool was used to annotate the whole mitochondrial genome [30,31]. Manual corrections were performed using MEGA11.0 and based on the mitochondrial genomes of related species [32]. A comparative circular genome map was generated using the BLAST Ring Image Generator [33]. The ratio of the nonsynonymous substitutions per nonsynonymous site (Ka) and synonymous substitutions per synonymous site (Ks) substitutions in 13 PCGs were calculated using the DnaSP5 software [34]. The SWISS-MODEL repository was used to analyze the tertiary structures of the proteins [35]. Webservice ESPript 3.0 was used to analyze the protein-coding sequence of nad4 [36].

### 2.3. Construction of Phylogenetic Trees

The phylogenetic tree was constructed using Cyphoderrinae Gorochov, 1988 (*Cyphoderris monstrosa* Uhler, 1864) and Prophalangopsinae Kirby, 1906 (*Tarragoilus diuturnus* Gorochov, 2001), as outgroups and 28 species of Anostostomatidae as ingroups (Table S2).

PhyloSuite v1.2.3 was used to generate four datasets [37]: (I) PCG123: PCGs with all three codon positions; (II) PCG12: PCGs with the first and second codon positions; (III) PCG123 + 2R: PCGs with all three codon positions and two rRNAs (12SrRNA and 16SrRNA); (IV) PCGAA: the amino acid sequence of a protein-coding gene. Dataset heterogeneity and substitution saturation were assessed using AliGROOVE v1.08 [38] and DNAMBE [39].

Multiple sequence alignments were performed using MAFFT v7.505 in PhyloSuite v1.2.3 in auto-mode [40]. The concatenated datasets were generated using PhyloSuite v.1.2.3, and the best-fit partition model was selected using ModelFinder v2.2.0 under the Bayesian information criterion to obtain the GTR + F + I + G4 model [41]. Bayesian inference (BI) phylogenies were constructed using MrBayes v3.2.7a [42]. Outgroups were manually selected; MCMC generations were set to 10,000,000; sampling frequency was 1000; four MCMC chains were run; and the Burnin Fraction value was 0.25. Maximum likelihood (ML) phylogenies were inferred using IQ-TREE v2.2.0 under the partition model for 1000 standard bootstraps and the Shimodaira–Hasegawa-like approximate likelihood-ratio test [43,44]. The resulting phylogenetic tree was visually edited using the online website iTOL and was presented as a diagram [45].

## 3. Results and Discussion

### 3.1. Mitochondrial Genome

**Figure 1:**
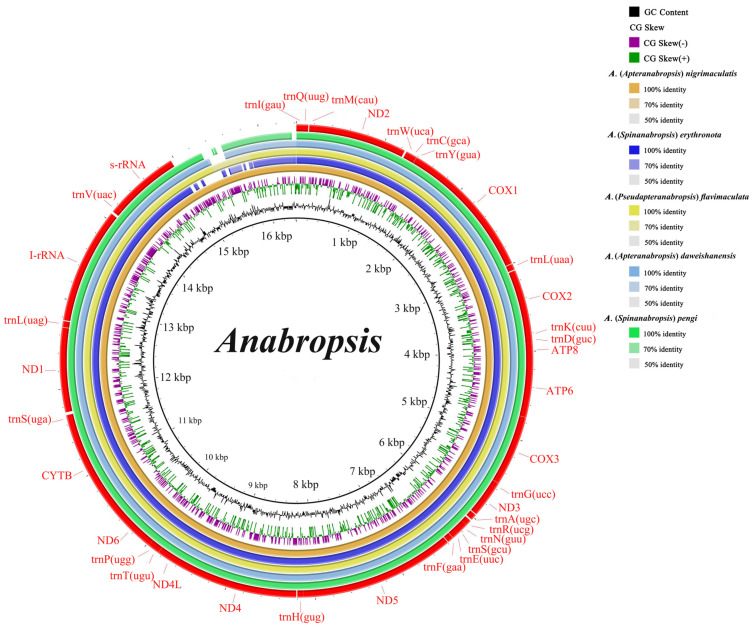
Comparative genomic circle map of five newly sequenced species. Note: The innermost layer was the self-proportional sequence of the reference genome (*Anabropsis* (*Pseudapteranabropsis*) *nigrimaculatis*, the second layer was GC content, and the third layer was CG-skew. The outer four-layer structure is consistent with the reference sequence, which consists of *A.* (*Spinanabropsis*) *erythronota*, *A.* (*Pseudapteranabropsis*) *flavimaculata*, *A.* (*Apteranabropsis*) *daweishanensis*, and *A.* (*Spinanabropsis*) *pengi* from inside to outside. Thirty-seven genes are situated at the outermost layers.

The five newly assembled mitochondrial genome sequences were all circular structures. The genomes ranged in size from 15,985 bp to 16,423 bp. The size difference was primarily due to the length of the control region (Figure 1). The genome has 37 typical mitochondrial genes, comprising 13 protein-coding genes, 22 tRNAs, and 2 rRNAs, with the same transcription direction. The gene order was consistent with that of the ancestral insect mitochondrial genome [26]. The start codons in all five species were ATN, and the end codons were mostly TAN, except for *cox2*, which ended in an incomplete T. Ojala et al. hypothesized that polycistronic pre-mRNA transcripts are processed by endonucleases that recognize the secondary structures of tRNAs, and that the polyadenylation of adjacent PCGs produces functional stop codons from partial stop codons, such as a single T-nucleotides [46,47]. Incomplete end codons are common in the mitochondrial genomes of many invertebrates and mammals [48,49,50,51].

The base content of the mitochondrial genomes from the five species showed a significant A + T bias, with A + T content ranging from 72.7% to 75.8%. The AT-skew was positive and the GC-skew was negative for the complete mitochondrial genome and third codon positions. Conversely, the AT-skew was negative and the GC-skew was positive for the PCGs, rRNAs, and first and second codon positions, indicating a higher frequency of T over A and G over C. These skew patterns may reflect a balance between mutational and selective pressures during replication [52].

### 3.2. Mitochondrial Gene Interval and Overlapping Regions

Intergenic spaces and overlapping regions were observed across the five genomes. The intergenic spacer regions ranged from 1 to 108 bp in size, with the maximum interval predominantly occurring between *trnS2* and *nad1*. The longest interval was 108 bp in *Anabropsis* (*Pseudapteranabropsis*) *flavimaculata*, followed by 55 bp in *Anabropsis* (*Spinanabropsis*) *erythronota*. All species had a 1 bp intergenic spacer between *nad5* and *trnH*. Long intergenic noncoding spacers (100–500 bp) have also been found in other orthopterans [20,53].

Overlapping regions varied in size from 1 to 23 bp. The largest overlap (23 bp) occurred between *trnL1* and *rrnL* in *Anabropsis* (*Spinanabropsis*) *erythronota*. This was followed by an overlapping region of 8 bp in five species, occurring between *trnaW* and *trnaC* and *trnaY* and *cox1*. Other notable overlaps included 7 bp between *nad4* and *nad4l*, 4 bp between *atp8* and *atp6*, and 1 bp between both *trnT* and *trnP* and *nad6* and *cytb*.

### 3.3. Protein Coding Genes and Codon Usages

**Figure 2:**
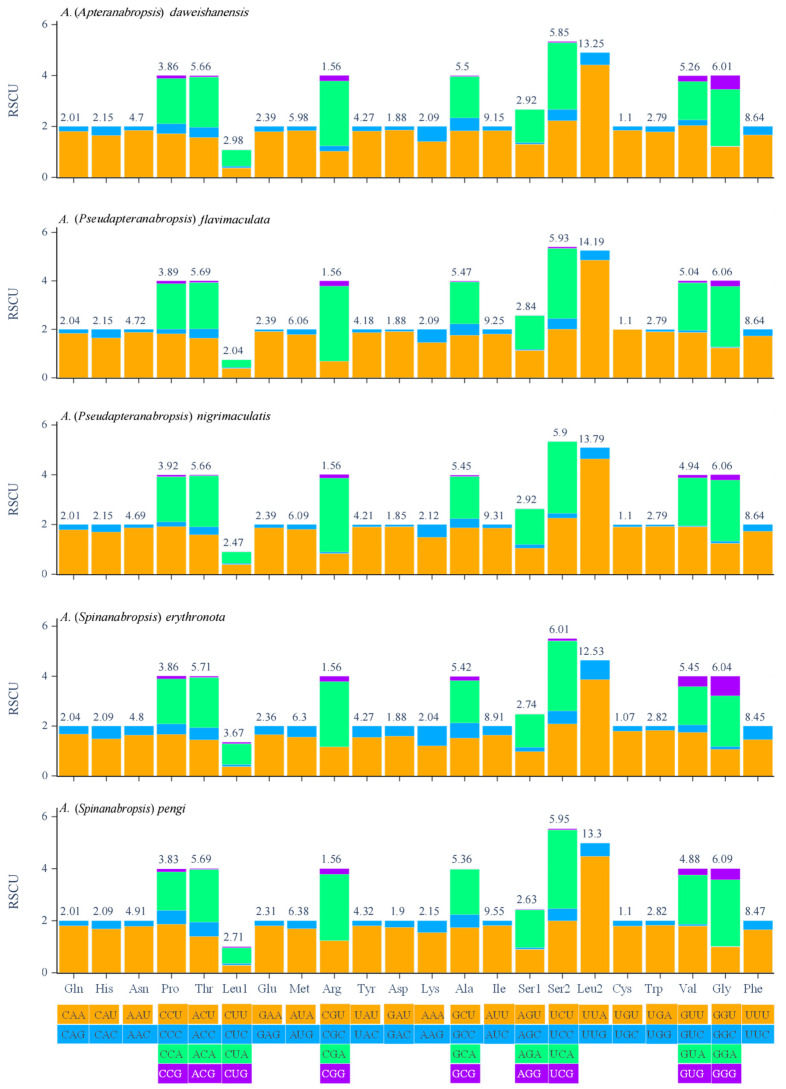
Relative synonymous codon usage (RSCU) of the mitogenome of five species.

The codon usage bias among PCGs was assessed by quantifying the relative synonymous codon usage (RSCU) values. RSCU measures the observed frequency of each codon relative to the expected frequency under equal codon usage. Analysis of the RSCU in the five mitochondrial genes revealed that Ser2 had the highest coding frequency, followed by Leu2, while Leu1 had the lowest coding frequency. Among the 62 amino acid codons, 33 were used less frequently (RSCU < 1) and 29 were used more frequently (RSCU > 1). In *Anabropsis* (*Apteranabropsis*) *daweishanensis*, 32 codons were used less frequently (RSCU < 1), and 30 codons were used more frequently (RSCU > 1; Figure 2). The UUA codon was the most frequently used.

### 3.4. The Rate of Evolution of 13 PCGs

**Figure 3:**
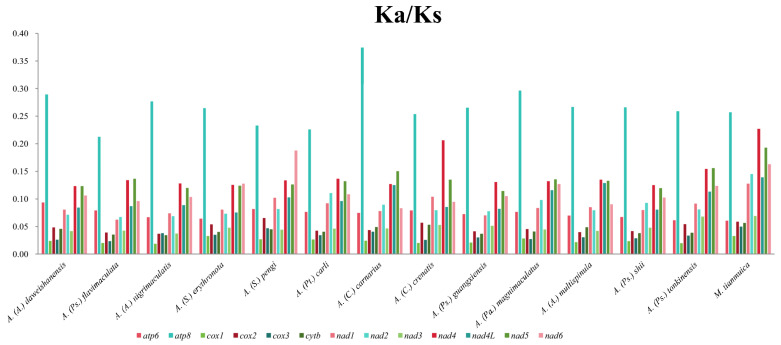
Ka/Ks values of 13 PCGs in 14 species of Anabropsinae.

We calculated the ratio of Ka and Ks across 14 Chinese Anabropsini species to estimate the evolutionary rate of PCGs. All the values were below 1, indicating that the genes were selected by purification (Figure 3). Among them, *cox1* exhibited the lowest Ka/Ks value, while *atp8* exhibited the highest. This result is consistent with the calculated results for alate and apterous species (Figures S1 and S2). When comparing the Ka/KS values of 13 PCGs in alate and apterous species, *atp8*, *cox1*, *cox3*, and *nad6* demonstrated higher values in apterous species than in alate species. Conversely, the values of other genes were lower in apterous species compared with alate species. However, there were no significant differences in the Ka/Ks values of any of the PCGs between alate and apterous species, indicating that their evolutionary rates are consistent. Therefore, further discussion is needed on the validity of classifying genera or subgenera based on wing length.

**Figure 4:**
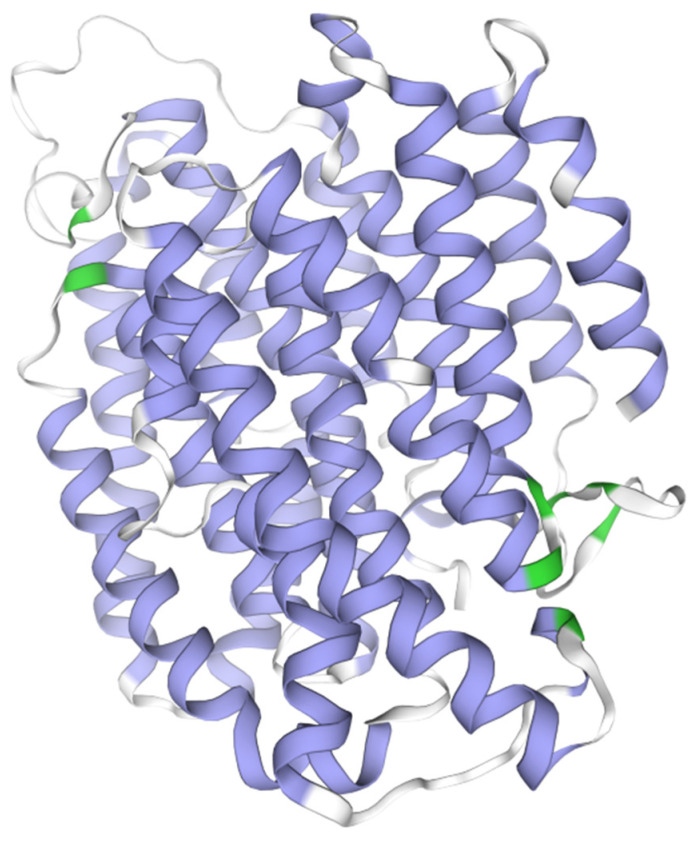
The protein tertiary structure of *nad4* of *Anabropsis* (*Carnabropsis*) *crenatis*. Purple is α-helix, green is β-sheet, and white is coil.

We selected *nad4*, which has a fast evolutionary rate, for protein sequence alignment (Figure 4). The comparative analysis revealed a high degree of sequence similarity in the *nad4* gene of Anabropsini, with 374 conserved sites, 71 variable sites, 16 parsimony-informative sites, and 55 singleton sites identified. These site-specific data provided crucial molecular evidence for elucidating the evolutionary patterns of the *nad4* gene within this tribe. Analysis of the predicted tertiary structure of *nad4* from the winged species *Anabropsis* (*Carnabropsis*) *crenatis* showed 21 α-helixes (purple) and 8 β-sheets (green). The variable sites were predominantly concentrated within the α-helix regions, with the highest density observed in the α14 (19 sites), α2 (11 sites), and α13 (11 sites) helices.

### 3.5. Substitution Saturation Tests and Nucleotide Heterogeneity

The third codon position is particularly susceptible to nucleotide composition bias and skew, potentially resulting in pseudogenes [54], or the source of most of the phylogenetic signals [55]. Due to its variability, it is essential to evaluate whether the datasets that include the third codon retain sufficient phylogenetic signal or require exclusion.

Substitution saturation was evaluated using the index of substitution saturation (Iss) for the PCG12 and PCG123 datasets across 30 species. In both datasets, the Iss was significantly lower than the critical Iss.c threshold, indicating a lack of saturation and a strong phylogenetic signal.

Pairwise comparisons of multiple sequence alignments were performed to evaluate heterogeneity in nucleotide divergence among datasets. The PCG12, PCG123 + 2R, and PCG123 datasets showed little heterogeneity (Figure S3). However, the heterogeneity of the PCGAA dataset was negative, suggesting poor phylogenetic value; therefore, it was excluded from subsequent tree construction (Figure S3).

### 3.6. Phylogenetic Analysis of Anostostomatidae

**Figure 5:**
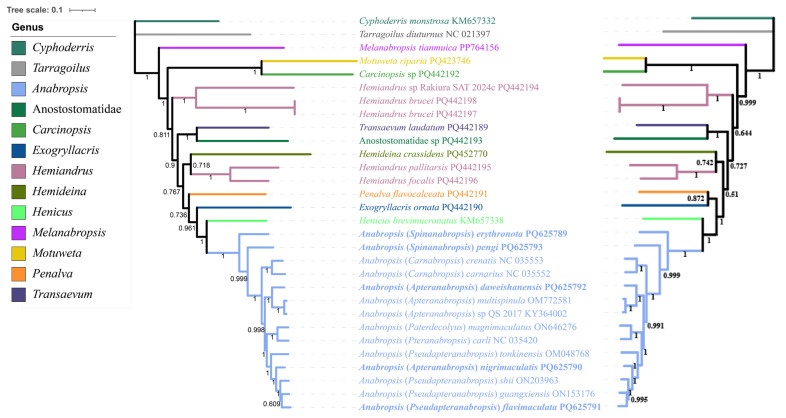
Phylogenetic tree obtained from BI analysis based on PCG123 and PCG123 + 2R, with the numbers on the branches indicating bootstrap supports. Note: The left is the topology of the PCG123 dataset. The right is the topology of the PCG123 + 2R dataset.

**Figure 6:**
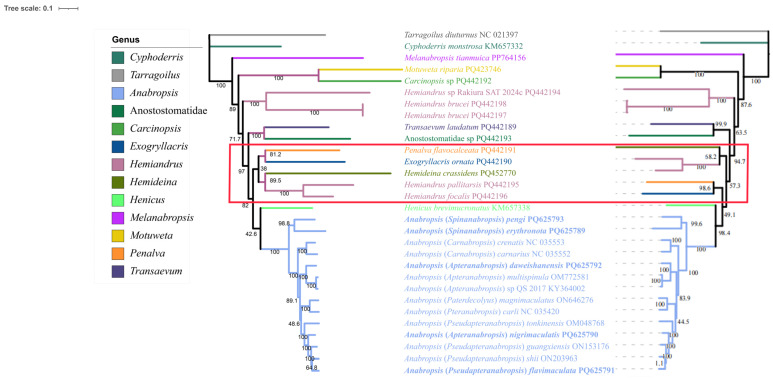
Phylogenetic tree obtained from ML analysis based on PCG123 and PCG123 + 2R, with the numbers on the branches indicating bootstrap supports. Note: The left is the topology of the PCG123 dataset. The topology of the PCG123 + 2R dataset is on the right. The red box shows the difference in the topology of the two datasets.

The phylogenetic tree built using the PCG123 dataset exhibited higher node confidence values than the phylogenetic tree constructed using the PCG123 + 2R dataset. The BI topology constructed using the PCG123 dataset showed a confidence value of 0.767 for (*Hemideina* + *Hemiandrus*) + (*Penalva* + (*Exogryllacris* + (*Anabropsis* + *Henicus*))) (Figure 5). The ML topology constructed using the PCG123 dataset showed a confidence value of 82 for ((*Exogryllacris* + *Penalva*) + (*Hemideina* + *Hemiandrus*)) + (*Anabropsis* + *Henicus*) (Figure 6).

All species within *Anabropsis* were clustered together. However, the genera *Melanabropsis*, *Exogryllaris*, and *Anabropsis*, all members of Anabropsini, did not form a monophyletic group. *Hemiandrus* was found to be paraphyletic, consistent with the findings of Trewick et al. [28]. *Anabropsis* was identified as the sister genus to *Henicus* brevimucronatus, a species from South Africa, supporting the results reported by Lu [28,30]. In contrast, *Henicus* and *Motuweta*, both classified within Anostostomatini, did not cluster as sister taxa.

The Anabropsini species from China did not cluster to form a monophyletic group. Notably, *Anabropsis* (*Apteranabropsis*) *nigrimaculatis* formed a cluster with other species within the *Pseudapteranabropsis* subgenus. Pang et al. originally classified *Pseudapteranabropsis* based on the male paraproctal outgrowth bifurcating in the lateral view, and described the female *Anabropsis* (*Apteranabropsis*) *nigrimaculatis* [6]. Xu and Shi later reclassified *Anabropsis* (*Apteranabropsis*) *nigrimaculatis* into *Apteranabropsis* based on male morphology [8]. Our results support retaining *Anabropsis* (*Apteranabropsis*) *nigrimaculatis* within the *Pseudapteranabropsis* subgenus.

Lu et al. reconstructed the phylogeny of six species from Anostostomatidae based on 13 PCGs. The relationship between subgenera within *Anabropsis* was proposed to be (*A.* (*Apteranabropsis*) + (*A.* (*Paterdecolyus*) + *A.* (*Pteranabropsis*)) [2]. These findings support a sister relationship between *Pteranabropsis* and *Apteranabropsis* [4]. In contrast, our expanded analysis based on BI and ML approaches demonstrated a different topology within the *Anabropsis* genus: ((*A.* (*Pseudapteranabropsis*) + (*A.* (*Paterdecolyus*) + *A.* (*Pteranabropsis*) + *A.* (*Apteranabropsis*) + *A.* (*Carnabropsis*) + *A.* (*Spinanabropsis*))). This study thus provides a novel perspective on phylogenetic relationships within *Anabropsis.* Our results contribute valuable insights into the evolutionary history of *Anabropsis* and establish a foundation for future comprehensive phylogenetic studies.

## 4. Conclusions

The five mitogenomes of Chinese *Anabropsis* species sequenced in this study demonstrated similar genome sizes, AT nucleotide bias, AT- and GC-skews, and codon usage of PCGs, aligning with previously reported mitogenomes from other orthopterans. Gene order remains highly conserved and identical to that of the putative ancestral insect. The Ka/Ks values for alate and apterous forms were not significantly different, suggesting that wing length may not be a reliable basis for dividing *Anabropsis* subgenera. Analysis of the protein tertiary structures demonstrated that the variable sites were predominantly located within the α-helix regions. Phylogenetic analyses using the PCG123 and PCG123 + 2R datasets suggest that the Chinese Anabropsini within Anostostomatidae are paraphyletic, whereas *Anabropsis* is monophyletic. Moreover, the topological structure of *Anabropsis* genera is stable. Mitogenome data demonstrated relationships among the major lineages of Anabropsini, and additional data may help further elucidate the relationships in this highly diverse lineage.

## Figures and Tables

**Table 1:** Voucher information of the specimens used for mitochondrial genome sequencing.

Specimens	Date of Collection	Collection Site	Longitude (E)	Latitude (N)	GB Numbers
*A.* (*Pseudapteranabropsis*) *nigrimaculatis*	2 August 2022	Maguan, Yunnan	104.0004	22.5115	PQ625790
*A.* (*Spinanabropsis*) *erythronota*	2 August 2022	Maguan, Yunnan	104.0004	22.5115	PQ625789
*A.* (*Pseudapteranabropsis*) *flavimaculata*	20 August 2022	Pinglong Mountain, Guangxi	109.8680	22.8403	PQ625791
*A.* (*Apteranabropsis*) *daweishanensis*	23 May 2021	Dawei Montain, Yunnan	101.5128	23.1467	PQ625792
*A.* (*Spinanabropsis*) *pengi*	13 August 2021	Yakou, Yunnan	99.1114	23.1722	PQ625793

## Data Availability

The five newly sequenced mitogenome sequences have been submitted to NCBI (Acc. Nos. PQ625789, PQ625790, PQ625791, PQ625792, and PQ625793).

## Supplementary Materials

The following supporting information can be downloaded at https://www.mdpi.com/article/10.3390/biology14070772/s1: Table S1. Basic genome information for five species was obtained in this study. Table S2. List of samples included in phylogenetic analysis. Figure S1. The Ka/Ks values of apterous species 13 PCGs in Anabropsini. Figure S2. The Ka/Ks values of alate species 13PCGs in Anabropsini. Figure S3. Heterogeneity test for different datasets. The average similarity score between the sequences is represented as colored squares, based on an AliGROOVE score ranging from −1 (indicating a large difference in rate from other datasets, red) to +1 (indicating the rate that matches all other comparisons, as in blue in this example) (Figure S4). Comparison diagram of *nad4* protein coding sequences of 14 species of *Anabropsis*. At the top is α-helix position and number in the protein secondary structure of *Anabropsis* (*Carnabropsis*) *crenatis*. The red background shows that the corresponding sites of the 14 sequences are highly consistent. The number represents the number of amino acids.
