# Supplementary material for: Comparative Analysis of the Mitochondrial Genomes of Five Species of Anabropsis (Orthoptera: Anostostomatidae) and the Phylogenetic Implications of Anostostomatidae
Source: Biology (Basel). 2025 Jun 26;14(7):772. doi: 10.3390/biology14070772 (PMC12292721; doi:10.3390/biology14070772)
Supplement: Supplementary file 1 [file biology-14-00772-s001.zip › biology-3664949-supplementary.pdf]

Table S1. Basic genome information for five species was obtained in this study.

Speceis	Base number	Reference genomes	Reference
Anabropsis (*Spinanabropsis*) *erythronota*	15985bp	NC_068726/ON153176	[4]
Anabropsis (*Spinanabropsis*) *pengi*	16081bp	NC_068726/ON153176	[4]
Anabropsis (*Pseudapteranabropsis*) *flavimaculata*	16364bp	NC_068726/ON153176	[4]
Anabropsis (*Pseudapteranabropsis*) *nigrimaculatis*	16423bp	NC_068726/ON153176	[4]
Anabropsis (*Apteranabropsis*) *daweishanensis*	16047bp	NC_068725/OM772581	[4]

Table S2. List of samples included in phylogenetic analysis.

Family	Subfamily	Genus	Species	Accession no.	Reference
Anostostomatidae	Anostostomatinae	*Deinacrida*	*Deinacrida connectens*	PQ442198	[28]
		Exogryllacris	*Exogryllacris ornata*	PQ442190	[28]
			Hemiandrus Rakiura	PQ442194	[28]
			Hemiandrus brucei	PQ442197	[28]
		Hemiandrus	*Hemiandrus focalis*	PQ442196	[28]
			Hemiandrus pallitarsis	PQ442195	[28]
			Hemiandrus sp.	PQ442193	[28]
		Hemideina	*Hemideina crassidens*	PQ452770	[28]
		Henicus	*Henicus brevimucronatus*	KM657338	[27]
		Motuweta	*Motuweta riparia*	PQ423746	[28]
		Penalva	*Penalva flavocalceata*	PQ442191	[28]
		Transaevum	*Transaevum laudatum*	PQ442189	[28]
	Anabropsini		*Anabropsis* (*Paterdecolyus*) *magnimaculatus*	KY364002	[3]
			Anabropsis (*Pteranabropsis*) *carli*	KY241792	Direct submission
			Anabropsis (*Carnabropsis*) *crenatis*	KY296453	Direct submission
			Anabropsis (*Carnabropsis*) *carnarius*	KY296452	Direct submission
			Anabropsis (*Apteranabropsis*) *multispinula*	OM772581	[4]
			Anabropsis (*Pseudapteranabropsis*) *shii*	ON203963	[4]
			Anabropsis (*Pseudapteranabropsis*) *guangxiensis*	ON153176	[4]
		Anabropsis	*Anabropsis* (*Pseudapteranabropsis*) *tonkinensis*	OM048768	[4]
			Anabropsis (*Apteranabropsis*) *nigrimaculatis*	PQ625790	This study
			Anabropsis (*Pseudapteranabropsis*) *flavimaculata*	PQ625791	This study
			Anabropsis (*Spinanabropsis*) *erythronota*	PQ625789	This study
			Anabropsis (*Spinanabropsis*) *pengi*	PQ625793	This study
			Anabropsis (*Apteranabropsis*) *daweishanensis*	PQ625792	This study
		Melanabropsis	*Melanabropsis tianmuica*	PP764156	Direct submission

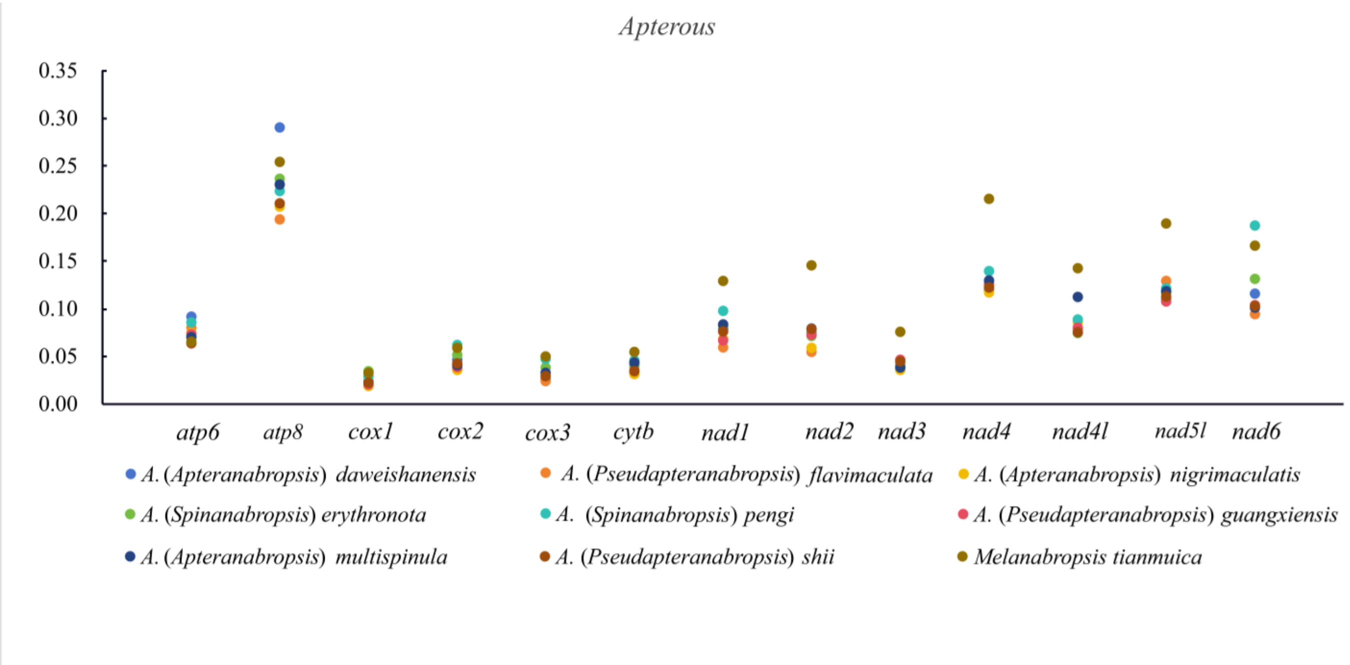

Figure S1. The Ka/Ks values of apterous species 13PCGs in the Anabropsini.

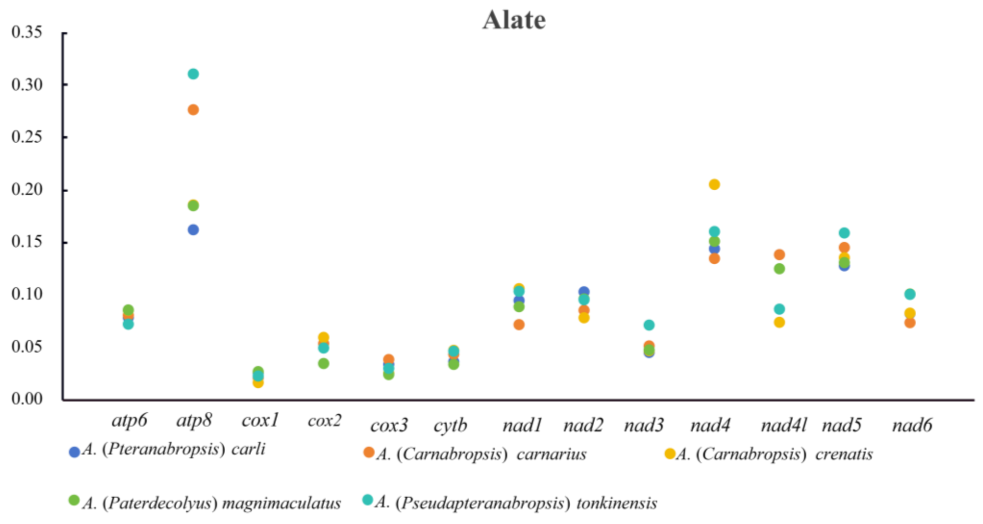

Figure S2. The Ka/Ks values of alate species 13PCGs in the Anabropsini.

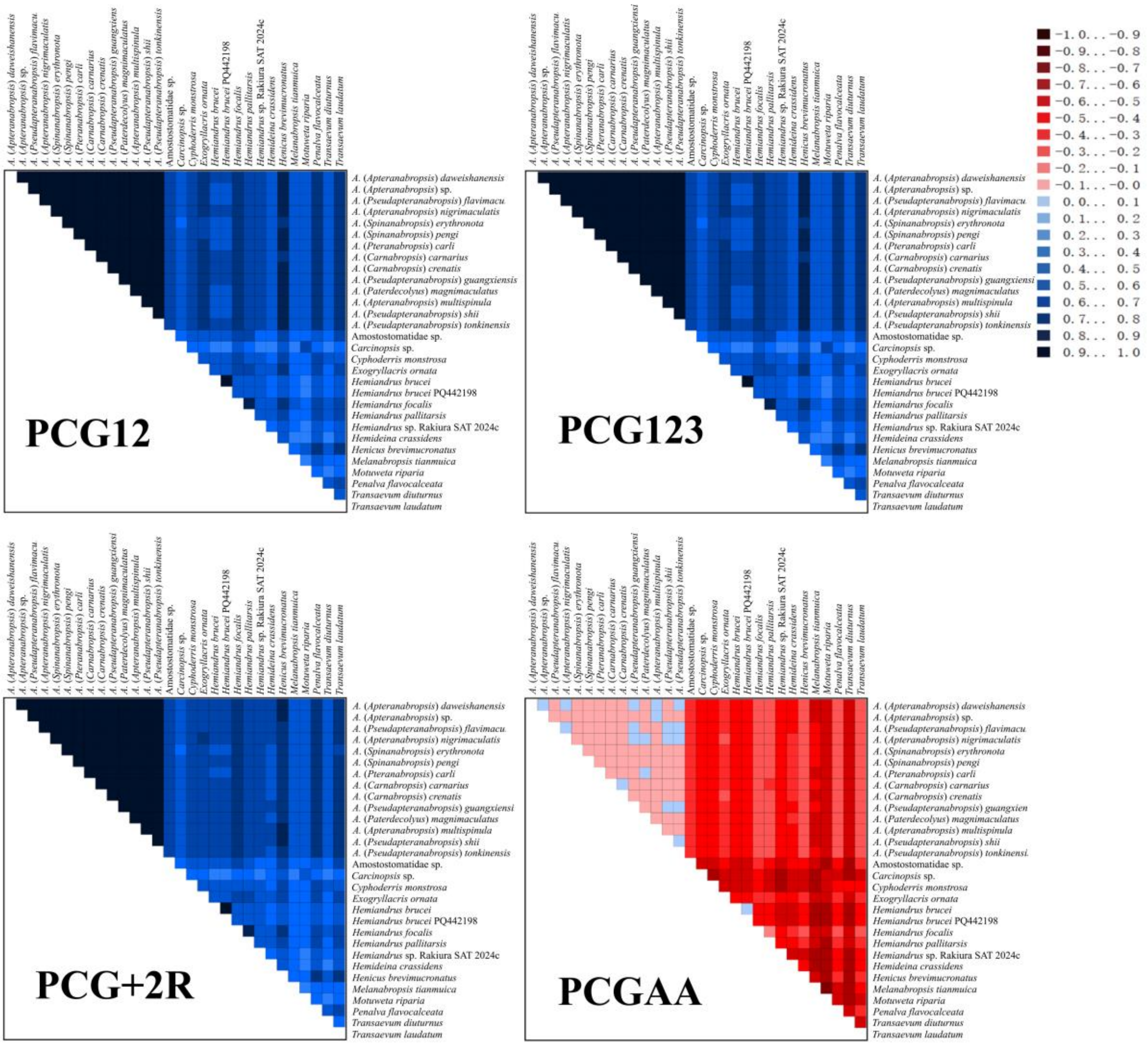

Figure S3. Heterogeneity test for different datasets. The average similarity score between the sequences is represented as colored squares, based on an AliGROOVE score ranging from -1 (indicating a large difference in rate from other datasets, red) to +1 (indicating the rate that matches all other comparisons, as in blue in this example).

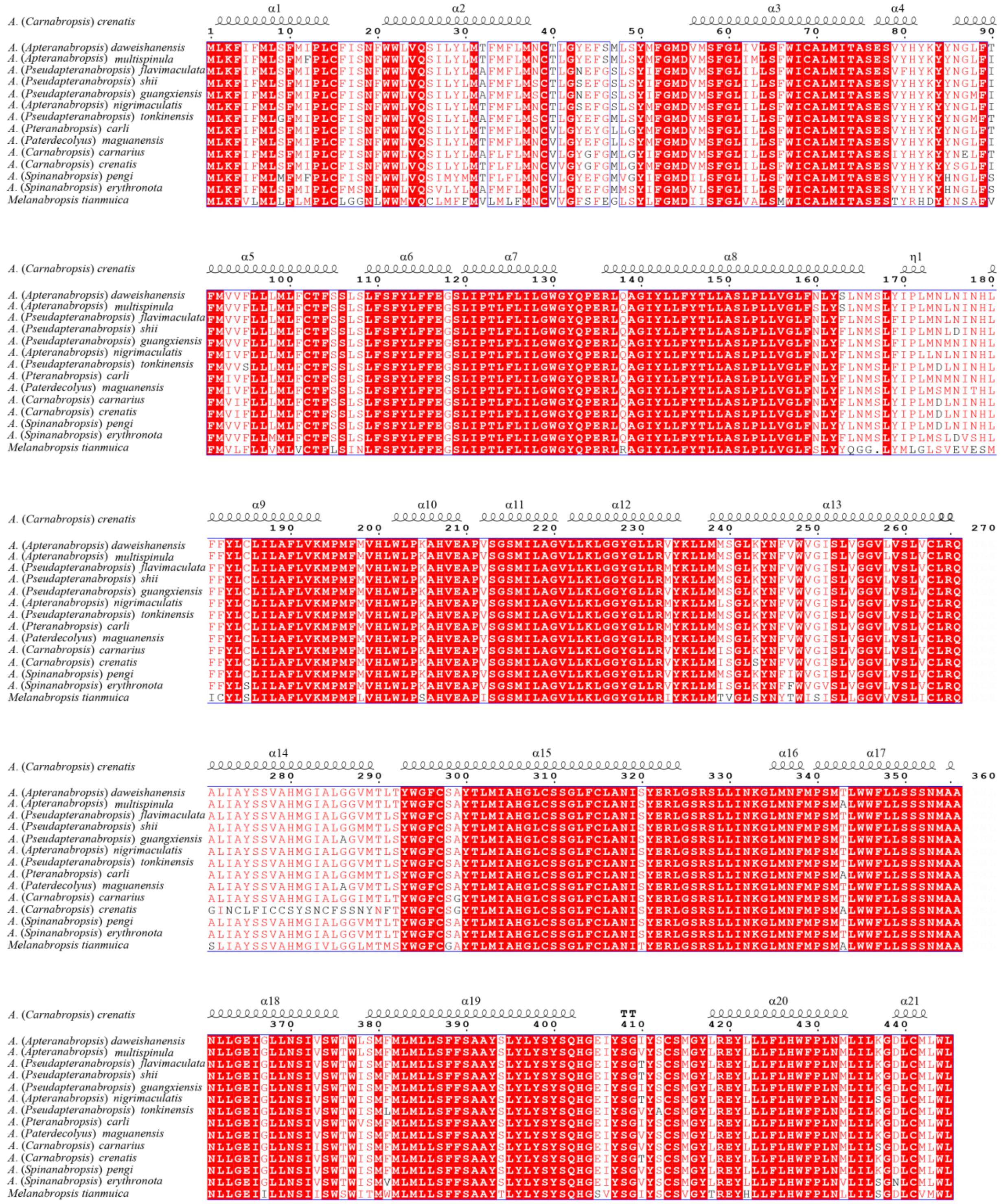

Figure S4. Comparison diagram of nad4 protein coding sequences of fourteen species of the *Anabropsis*. At the top is α-helix position and number of in the protein secondary structure of *Anabropsis* (*Carnabropsis*) *crenatis*. The red background shows that the corresponding sites of the 14 sequences are highly consistent. The number represents the number of amino acids.
